# A global palaeo-science community: an interview with Pal(a)eoPERCS

**DOI:** 10.1038/s42003-022-03936-2

**Published:** 2022-09-16

## Abstract

Out of the ashes departmental seminar breakdowns during the COVID-19 pandemic rose Pal(a)eoPERCS, a global effort to engage early career researchers from around the world in an accessible and productive community. We spoke to the committee of Pal(a)eoPERCS about the formation, success, and drive for their initiative.

**Rehemat Bhatia:** Pal(a)eoPERCS Funding Officer

**Jana Burke:** Pal(a)eoPERCS social media and advertising officer

**Pedro Godoy:** Pal(a)eoPERCS video editor

**Natasha Sekhon:** Pal(a)eoPERCS Website Manager

**Chrissy Hall:** Pal(a)eoPERCS scheduler

**Elizabeth Sibert:** Pal(a)eoPERCS Email list manager and general “catch-all”

Hi team, thanks for chatting today! Can you briefly introduce yourselves, your careers, and Pal(a)eoPERCS?

**Rehemat Bhatia (RB):** Hi! I am Rehemat. I am a geochemist and micropalaeontologist by training. I did my undergrad at Royal Holloway (University of London) (UK) and my PhD at University College London (UK). My PhD and subsequent postdoctoral research focused on using the trace element and isotope geochemistry of Cenozoic planktonic foraminifera to understand more about their palaeoecologies and responses to dramatic climate change intervals. After my postdoc at the University of Bristol (UK) ended, I switched career direction and now work outside of academia, but I still take part in a few academia-tied initiatives in my spare time - like Pal(a)eoPERCS! On the Pal(a)eoPERCS Committee I currently lead on putting together our funding bids and searching for financial support avenues. I am also part of the Earth Science Women’s Network’s Associate Board of Directors as their Co-Chair of Member Events, and regularly participate in science outreach initiatives. I love palaeo-science and really can’t imagine my life without it!

**Jana Burke (JB):** Hi, I am Jana. I am a micropaleontologist and paleoceanographer working primarily with planktonic foraminifera, based at Michigan State University. I am particularly interested in the ways that individual organisms, species, and communities change in response to changes in their environment, which is probably a good thing given the transitory nature of ECR life. I first fell in love with the pal(a)eo-sciences as an undergraduate at Smith College and recently completed my PhD at Yale University. I am passionate about contributing to a strong, supportive, inclusive community of pal(a)eo-ECRs, and also am a fan of wise-cracking, handicrafts, and scream-singing at karaoke.

**Pedro Godoy (PG):** Hi, I am Pedro, a vertebrate paleontologist and evolutionary biologist interested in systematics and macroevolution of tetrapods. I did my undergrad and MSc in my native Brazil. During my PhD at the University of Birmingham (UK), my research focused on macroevolutionary patterns during the evolutionary history of Crocodylomorpha. Following that, I did two postdocs, one in the US and another one in Brazil. On the Pal(a)eoPERCS Committee, I am responsible for the video editing and for uploading the recorded talks to our YouTube channel. Besides that, I am also the current Phylogenetics Editor of the Journal of Vertebrate Paleontology.

**Chrissy Hall (CH):** Hi, I am Chrissy. I am an invertebrate paleontologist interested in how life has responded to changing environments in the past. I did my undergraduate at the College of William and Mary and my MS and PhD at the University of California, Riverside. My master’s research was on Ediacaran fossils with three-fold symmetry, but since then my focus has shifted to ostracodes (small, bivalved crustaceans) from different times of climatic changes throughout the Cenozoic. I’ve been working on these questions as a postdoc at the University of Connecticut and the University of Haifa, and I am starting a visiting assistant professor at Lafayette College in the fall. On the Pal(a)eoPERCS Committee, I am responsible for scheduling the speakers we decide to invite and keeping our schedule up-to-date.

**Natasha Sekhon (NS):** Hi! I am Natasha, I am a speleothem scientist and karst hydrologist. I use the geochemical proxies of stalagmite (calcite deposits in caves) to discern past climate change. I did my undergraduate at the University of California, Irvine. My PhD at the University of Texas, Austin, and current postdoctoral research at Brown University focuses on piecing together extreme climates such as flooding events through the Holocene in the United States and the Philippines. I have the great pleasure and feel very fortunate that I get to explore caves in remote parts of the world. During these field campaigns, we set up instruments in caves to monitor the geochemical variability of cave dripwaters, which help inform our paleoclimate interpretations. On the Pal(a)eoPERCS committee, I am responsible for the upkeep and maintenance of the Pal(a)eoPERCS website.

**Elizabeth Sibert (ES):** Hi! I am Elizabeth. I am a micropaleontologist and oceanographer, and I am interested in how marine ecosystems respond to global change. I specialize in ichthyoliths, microfossil fish teeth, and shark scales, preserved mostly in deep-sea sediments, and use these tiny fossils to reconstruct fish and shark evolution and their roles in marine ecosystems on long timescales. My work is inherently interdisciplinary, using geological tools to address fundamental questions in biology and oceanography. I did my undergraduate in biology at University of California San Diego, and my MS and PhD in oceanography from Scripps Institution of Oceanography. My first postdoctoral position was as a Junior Fellow in the Harvard Society of Fellows at Harvard University, and I am currently a Hutchinson Postdoctoral Fellow through the Yale Institute of Biospheric Studies at Yale University. On the Pal(a)eoPERCS Committee, I manage the email list and send out twice-weekly seminar announcements. I also serve as the “catch-all”, develop leadership team meeting agendas, and keep track of all ongoing projects.

**Pal(a)eoPERCS Committee (PC; Figure** 1):The original leadership team for Pal(a)eoPERCS also included Andy Fraass, now an Assistant Professor at the University of Victoria, and Catherine Davis, now an Assistant Professor at North Carolina State University, both of whom contributed considerably to the initial phases of this series. Our current committee member, Jana Burke, of Michigan State University, is currently on family leave.

**Fig. 1:**
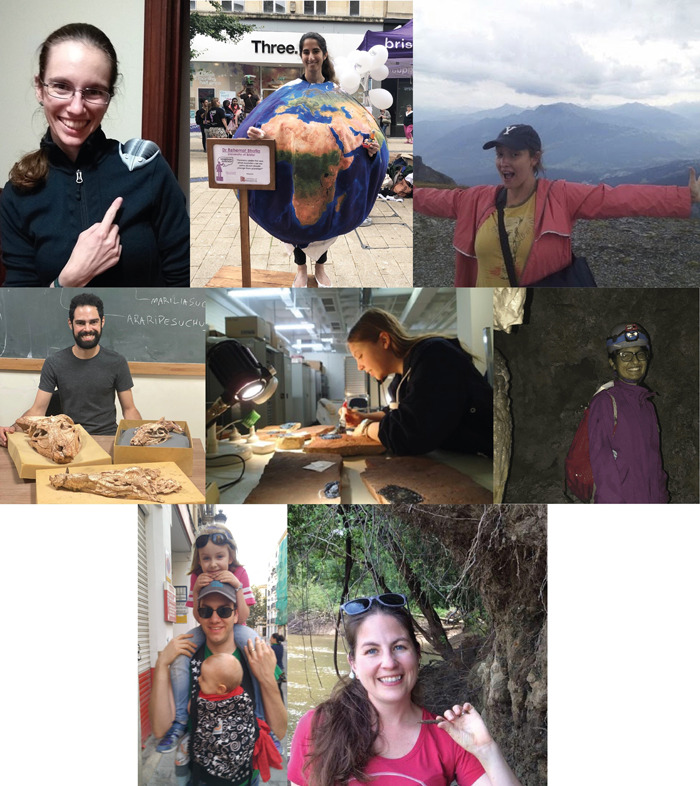
Image is a collage of images of 8 people, organized into 2 connected rows of 3 images and a third row of 2 images. The first 6 images are of current steering committee members. The top left image is a headshot of Elizabeth Sibert, a white woman with brown hair in a ponytail, wearing glasses. She is wearing a black jacket and has a gray 3D printed trilobite on her shoulder, which she is pointing to. The top middle image is a picture of Rehemat Bhatia, a brown-skinned woman, wearing a globe costume depicting the Earth. Her hair is tied back in a ponytail, and she is standing on a wooden box in a pedestrianised shopping area. The top right image is a photo of Jana Burke, a white woman wearing a baseball hat and a pink jacket on a mountain in Flims, Switzerland. Her arms are outstretched and she is grinning, with clouds and mountains in the background. The second-row left image is a picture of Pedro Godoy, a brown-eyed, dark-haired, and bearded man, wearing a dark gray T-shirt. He is smiling and sitting in front of a blackboard, on which the names of two crocodylomorph species can be read. In front of him, on a wooden table, there are three skulls of fossil crocodylomorphs. The second-row middle image is of Chrissy Hall, a white woman wearing a dark jacket. She is in a museum collections room painting liquid latex onto fossils, using a lamp to help them dry. The second-row right image is Natasha Sekhon, a brown-skinned woman, wearing glasses and in a pink rain jacket, brown pants with a red backpack. She is wearing a cave helmet with two headlamps and nitrile gloves. She is standing inside a cave in south-eastern New Mexico. The lower images are of former committee members. The lower left-hand image is of Andy Fraass, a white-skinned man, wearing a green shirt, grey hat, sunglasses, and a wedding band. He has a white-skinned young girl on his shoulders in a pink t-shirt with two white stripes on the sleeves and jeans. He also has a white-skinned baby strapped to his chest in a black and white child carrier. They are walking on a sidewalk in a city. The lower right-hand image is of Catherine Davis, a white woman with brown hair, wearing a red shirt with sunglasses on her head. She is standing on a muddy bank of the PeeDee River holding a belemnite and smiling. Pal(a)eoPERCS

For those who may not have heard of Pal(a)eoPERCS, what is it and what does it aim to do for the palaeontological community?

**PC:** Pal(a)eoPERCS (**P**al(a)eo **E**a**R**ly **C**areer **S**eminars) is a weekly, virtual, inclusive, accessible, institution-independent, global seminar series featuring Early Career Researchers (ECRs) in all fields of pal(a)eo-sciences. We launched the series in June 2020 in response to the COVID-19 pandemic, and have had an incredibly positive response from the palaeo community, with over 1900 sign-ups to our mailing list, > 2000 followers on Twitter, >100 followers on Instagram and a live, weekly, audience attendance of 30–60 individuals from all career stages, palaeo-disciplines and career paths worldwide, with another 50–200 individuals watching seminars asynchronously. We would also like to make it clear that Pal(a)eoPERCS is an independent endeavour and does not reflect the views of our respective affiliated organisations, institutions, and financial supporters.

We are an international, institution-independent coalition of early career researchers and professionals, and are highly committed to showcasing the wide diversity in our community. We actively ensure diverse speaker demographics, with a specific focus on the participation of historically excluded groups in the earth, marine and environmental sciences (e.g., those who identify as women, ethnic minorities, having a disability, LGBTQIA+, first generation, from a low-income background, etc.). We actively work to ensure a geographically diverse speaker line-up, and regularly feature ECRs based at institutions from all continents—the only continent we have yet to feature speakers from is Antarctica. Whilst we started unfunded and grassroots, we are now financially supported by six international learned societies and groups. The funding enables us to maintain a digital presence, provide speaker honoraria, and improve accessibility of the seminar series.

The remit of the series is interdisciplinary across the palaeo-sciences, from analytical (geo)chemistry to palaeobiology to palaeoclimate to palaeoceanography - essentially any research that involves palaeo-science or interacts with it (e.g., archaeology). We also host seminars which are on related topics such as decolonising (palaeo)-science and open access, and recently launched a special series of seminars focusing on Inclusive Fieldwork.

Seminars feature a 30 min talk, short Q&A, and informal speaker “tea-time” to foster collaboration and networking. Tea-time is a particularly novel feature of our series, and we know from participant feedback that new collaborations have formed as a result of our networking provisions. Seminars are open to those from all stages and workspaces, but feature ECR speakers specifically: those in the early stages of building their career and networks, on precarious contracts, and most affected by the ongoing COVID-19 pandemic. To date, we have hosted 95 seminars featuring speakers worldwide.

We also provide an asynchronous participation option recording most seminars and providing access either for a limited period of time or permanently (following speaker preferences) for those who cannot join in real-time. We also implement auto-captioning as standard at all seminars, and encourage our speakers to use colour-blind friendly graphics in their presentations (and provide resources to help). If particular accessibility requirements are requested from speakers and/or attendees we work with them to find an option that best supports their participation. We advertise the series to universities and learned societies globally, and international earth science-focused mailing lists. We undertake further advertising before the start of academic terms (ensuring our partnerships continue), and leverage social media to connect with the wider palaeo-community. Evaluation and regular refinement and monitoring of our practices is also undertaken on a regular basis to ensure we are tailoring the series to community needs.

What inspired the creation of Pal(a)eoPERCS?

**ES:** Pal(a)eoPERCS was initially conceived in response to departmental seminars being completely halted due to the onset of the COVID-19 pandemic in spring 2020. Departmental seminars, particularly those which feature speakers from outside the institution, are one of the main ways that intellectual ideas are exchanged. With the onset of the pandemic, seminars completely halted, leaving an intellectual void that left many folks feeling very isolated. We started Pal(a)eoPERCS to fill this void. The early-career researcher focus was chosen because ECRs are often overlooked when scheduling departmental seminars, but are often doing cutting edge and exceptional research. ECRs were particularly hard hit by the isolation of the pandemic, as their professional networks are less developed, so by focusing on ECRs, we were able to both feature exceptional research, while also providing valuable professional development and networking opportunities for the next generation of researchers. We also hoped that by opening Pal(a)eoPERCS to all fields of paleo-science (rather than focusing on just paleoclimate or paleontology, for example), we could create a community which was broader-thinking and synthesized across a wide range of topics.

Since Pal(a)eoPERCS is, by definition, a virtual seminar, we decided early on to have a global scope—anyone with an internet access who is interested can participate. We also made the choice to include and feature researchers from around the world. Finally, we built Pal(a)eoPERCS with digital accessibility and inclusion at the forefront, with the aim of building a fully accessible, global, and mutually supportive community of ECRs studying the processes that shape our planet and the life on it.

What are some of your highlights from the series so far?

**RB:** During August 2021, we received our first financial support offer from the Geological Society of London.

**CH:** We recently scheduled our 100th speaker! We have had speakers from 21 countries, from every continent except Antarctica.

**NS:** Our website has seen a steady increase in visits over the two years since the start of Pal(a)eoPERCS. May 2022 saw all-time views reaching close to 10,000.

**PG**: We had some cool special events, such as the collaborative seminars with GeoLatinas (https://geolatinas.org/) and the team JOIDES Resolution (which showed us what is done on board during a field trip at sea). We also had a special discussion panel on “Navigating Grad School” and will hold a special series on Inclusive Fieldwork.

**ES:** It is really rewarding to see such a diverse group of seminar presenters and attendees, and to watch the community grow - our email list surpassed 1900 people recently!. It is also wonderful to work with this exceptional leadership team to build and expand this series, build connections, and serve the community. Finally, I really love learning about something entirely new to me every week!

**PC:** We’ve had a lot of great support from both junior and senior folks on social media and through our feedback forms. One real highlight was seeing a mention in Roy Plotnick’s new book “Explorers of Deep Time: Paleontologists and the History of Life”. It really made our week when we found out!

The field has a historical record of poor representation, diversity, and opportunities for people with many different identities and backgrounds. Do you think there is progress being made in welcoming and promoting a diverse research environment?

**ES**: I think one thing that has been particularly rewarding about Pal(a)eoPERCS has been working behind the scenes to try to build something that encompasses the ideals of promoting a welcoming and diverse research environment. We’re a tiny drop in the bucket, and we are far from perfect, but I do think that the needle is inching towards progress, and it is nice to be a small part of that.

**RB**: Another way that we are actively advocating for equitable progress is through our involvement with the revision of the 500 Women Scientists Inclusive Meetings Guide (see article here). It is so exciting to be able to contribute toward this initiative, and have the opportunity to share good practices with the wider STEM community using the lessons we have learned over the past two years. We’re all really enjoying participating, and hope that the guide has a positive effect with a wide-reaching impact.

**PC:** We also really hope that by elevating ECRs and offering speaking, networking, and development opportunities needed for career progression (in any sector), that we can support efforts to and contribute towards making our community more equitable, diverse, inclusive, and accessible and help to retain those underrepresented in our field. We also try incredibly hard to promote an inclusive and nurturing environment at every seminar session (e.g., Code of Conduct implementation, anonymous question submissions, and moderation) to ensure all involved—speakers and attendees—can get as much out of participating as possible, are able to ask questions freely and feel that our virtual seminar space is one that is safe.

How can individuals get involved in Pal(a)eoPERCS? Who is welcome to join in?

**PC:** We encourage everyone who is interested in or actively doing research in the fields of Palaeo- to attend Pal(a)PERCS seminars. We have a ‘participate’ option through which participants join a mailing list, contingent on reading and agreeing to our code of conduct. Through our mailing lists, we send out notifications for the upcoming seminars. Anyone from any career stage or path who is interested in palaeo-sciences is welcome to join our mailing list and attend seminars: https://paleopercs.com/participate/. You can also find us on Twitter and Instagram @PalaeoPERCS.

The interaction between the broad fields of Palaeo during our seminar stimulates great conversations and discussions and we all definitely learn something new every week too!

As for speakers, our goal is to highlight the work of early career researchers in Palaeo. Our website has permanent links where we invite nominations for potential speakers: https://paleopercs.com/nominate-speakers/. We encourage folks to self-nominate as well!

Pal(a)eoPERCS has received a lot of support from learned societies and groups. Would you like to acknowledge their contributions?

**PC:** We are incredibly thankful to be financially supported by The Royal Society of Chemistry, The Geochemistry Group, The Geological Society of London, The Cushman Foundation for Foraminiferal Research, The Paleontological Society, and The Quaternary Research Association. We are currently making a financial plan for 2023, and plan to reach out to other learned societies later this year to secure more funding. We also would like to say a big thank you to everyone —both inside and outside academia - for spreading the word about the series. The response has been so encouraging, and the ongoing and increasing support really is a positive for us. We are incredibly proud to have created an inclusive initiative that is highly valued by the palaeo community, has supported many early career individuals at what continues to be a challenging time in their lives, and are excited to see how it evolves in the future.

*This interview was conducted by Senior Editor Luke R. Grinham*.

